# Green synthesis of Ag NPs on magnetic polyallylamine decorated g-C_3_N_4_ by *Heracleum persicum* extract: efficient catalyst for reduction of dyes

**DOI:** 10.1038/s41598-020-63756-4

**Published:** 2020-04-20

**Authors:** Pourya Mohammadi, Majid M. Heravi, Samahe Sadjadi

**Affiliations:** 1 Department of Chemistry, School of Science, Alzahra University, PO Box 1993891176, Vanak, Tehran Iran; 2 Gas Conversion Department, Faculty of Petrochemicals, Iran polymer and Petrochemicals Institute, 15 km Tehran-Karaj Highway, Pajuhesh Science and Technology Park, Pajuhesh Boulevard, postal cod; 14977-13115, PO Box 14975-112, Tehran, Iran

**Keywords:** Chemistry, Catalysis, Environmental chemistry

## Abstract

Silver nanoparticles were immobilized on magnetic polyallylamine (PAA) decorated g-C_3_N_4_ by using *Heracleum persicum* extract as a biological reducing and stabilizing agent. The resulting nanocomposite, Fe_3_O_4_-g-C_3_N_4_-TCT-PAA-Ag, was then characterized using BET, VSM, XRD, TGA, FTIR, TEM, EDS and ICP. The catalytic performance of the synthesized nanocatalyst was considered in the reduction of rhodamine B, and methyl orange in the presence of sodium borohydride in the aqueous medium at room temperature. The results showed that Fe_3_O_4_-g-C_3_N_4_-TCT-PAA-Ag nanocomposite could promote both reduction reactions efficiently in very short reaction times (70–100 s). In addition, Fe_3_O_4_-g-C_3_N_4_-TCT-PAA-Ag could be magnetically recovered and recycled for several cycles with no significant decrease in its catalytic performance. Using the experimental results, the rate constant, enthalpy, and entropy of the reduction reactions of both dyes were estimated.

## Introduction

Recently, significant consideration has been attracted to the environmental challenges related to water treatment^1^. The wastewater of the textile industry that contains aromatic dyes with low biodegradability is increasing, creating one of the principal sources of serious water contamination^2–4^. These industries evacuate sewage into the water; several of them are carcinogenic and mutagenic to humans^5^. Discharged dyes can also undergo imperfect anaerobic degradation, causing extra toxicity induced by the final products. In addition, coloration reduces sunlight infiltration and oxygen dissolution in water, which is also a serve menace to the aquatic ecosystem^6^. The malapropos use of dangerous chemicals in the textile water causes some serious influence on the safety and health. Ulcers, chemical burns, skin diseases, irritation, and respiratory difficulties are prevalent amongst workers in water treatment factories^7^.

Methyl orange (MO) as an organic azo dye, is toxic dye that is broadly utilized as a chemical reagent in food, paper, leather, and textiles industries. Recently, it has been applied as a natural dye in several industries, and commonly discharged without additional treatment in the ecosystem. This topic has a high threat to aquatic and animal life and possesses a direct influence on human health^8^.

Rhodamine B (RhB), as a nitrogen-containing cationic dye, is broadly utilized in foodstuffs, and textiles, as well as a tracer fluorescent^9^. This dye can undergo reductive anaerobic degradation and produced carcinogenic aromatic amines^10^. It has been experimentally proven that RhB causes inflammation of the skin, eyes, and respiratory tract.

Different biological, chemical, and physical, treatment methods have been developed over the last years for the treatment of wastewater. Among them, applying sodium borohydride as a reducing agent is an effective way to reduce dyes to less harmful compounds^11^. Metal nanoparticles as a catalyst were used in dye reduction due to their capability to provide higher specific surface areas to hasten these reactions^12^. Among them, silver nanoparticles (Ag NPs) due to their unique physicochemical features, are broadly utilized in various fields such as health, medicine, animal husbandry, agriculture, household, packaging, and electronics^13^. One of the main problems in the application of nanoparticles in reactions is relevant to their agglomeration. Immobilization of NPs on suitable supports overcomes the problems regarding their separation, stability, and recovery. In this context, several supports such as graphene oxide, TiO_2_, zeolite, and Fe_3_O_4_ have been applied for the immobilization of nanoparticles^14–17^. Among various supports, g-C_3_N_4_ displays good chemical stability^18–20^. g-C_3_N_4_ provides more applications than carbon materials due to the incorporation of nitrogen atoms in the carbon structure that can have great versatility and simplicity in designing and enhancement of its chemical, catalytically, electrical, and optical properties^21–24^.

**Figure 1:**
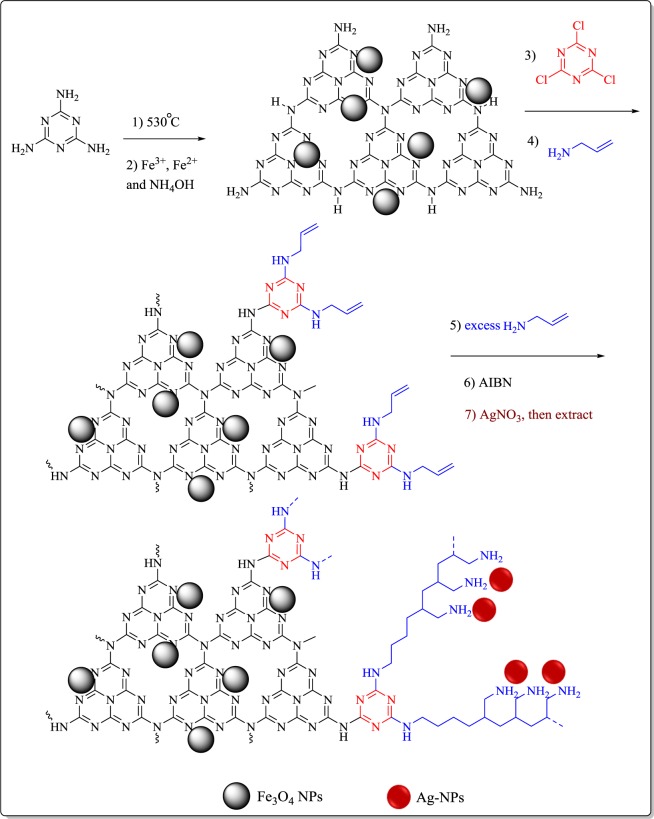
Preparation of the Fe_3_O_4_-g-C_3_N_4_-TCT-PAA-Ag nanocomposite.

In continuation of our research on the heterogeneous catalyst^25–28^, recently we disclosed the catalytic utility of the g-C_3_N_4_ hybrids and composites^29–33^. Inspired by the promising results of these hybrids/composites, in the present study, silver nanoparticles were synthesized using *Heracleum persicum* extract as the reducing and stabilizing agent. These nanoparticles were immobilized on the magnetic polyallylamine decorated g-C_3_N_4_ substrate (Fe_3_O_4_-g-C_3_N_4_-TCT-PAA-Ag) (Fig. 1). The catalytic activity of the Fe_3_O_4_-g-C_3_N_4_-TCT-PAA-Ag nanocomposite is investigated in the reduction of rhodamine B, and methyl orange dyes and the kinetic and thermodynamic terms of these reactions were also discussed. Moreover, the recyclability of the nanocatalyst for both reduction reactions is investigated.

## Result and Discussion

### Characterization of the as-prepared nanocomposite

**Figure 2:**
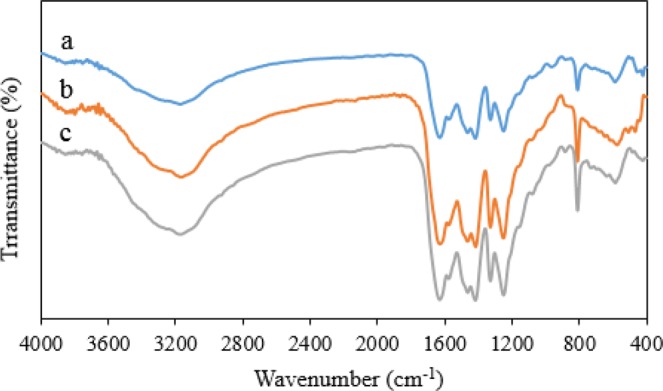
FTIR spectra of (**a**) Fe_3_O_4_-g-C_3_N_4_, (**b**) Fe_3_O_4_-g-C_3_N_4_-polyallylamine, and (**c**) Fe_3_O_4_-g-C_3_N_4_-TCT-PAA-Ag nanocomposite.

FTIR analyses were carried out to get reliable information about the functional group in Fe_3_O_4_-g-C_3_N_4_-TCT-PAA-Ag nanocatalyst. In Fig. 2a, FTIR spectrum of Fe_3_O_4_-g-C_3_N_4_, the strong absorption peak at 588 cm^−1^ was related to the stretching vibration the Fe-O bond. The strong absorption peak that appeared at 804 cm^−1^ corresponded to the bending vibration of the s-triazine ring. The absorption bands at 1200–1400 cm^−1^ were due to types of C-N stretching vibration mode. The peak present at the region 1621 cm^−1^ was due to C=N stretching vibration mode. In addition, the peaks at 3200–3500 cm^−1^ were due to NH, and OH stretching vibration mode. It, therefore, confirmed the presence of Fe_3_O_4_ and g-C_3_N_4_ structures^34^.

Figure 2b showed the FTIR spectrum of the Fe_3_O_4_-g-C_3_N_4_-polyallylamine nanostructure. When the polymer was connected to Fe_3_O_4_-g-C_3_N_4_, the peak intensity was increased in the NH region and on the other hand, a weak peak appeared in the 2929 cm^−1^ region, which may be related to the stretching vibration of the C-H bond of the polymer chain.

The FTIR spectrum of Fe_3_O_4_-g-C_3_N_4_-TCT-PAA-Ag was shown in Fig. 2c. It could be seen from the FTIR spectrum of Fe_3_O_4_-g-C_3_N_4_-TCT-PAA-Ag that after the deposition with Ag NPs on Fe_3_O_4_-g-C_3_N_4_-Polyallylamine, there was no change in the FTIR spectrum of the prepared nanocomposite, indicating that the Fe_3_O_4_-g-C_3_N_4_-Polyallylamine remained stable during the synthesis of the silver nanoparticles.

**Figure 3:**
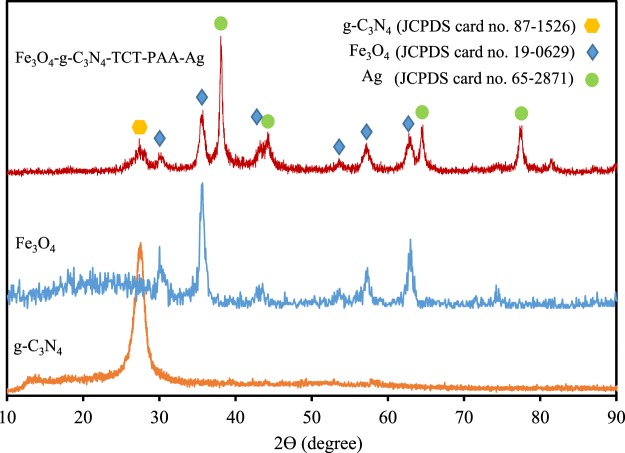
XRD patterns of Fe_3_O_4,_ g-C_3_N_4_ and Fe_3_O_4_-g-C_3_N_4_-TCT-PAA-Ag nanocomposite.

Figure 3 exhibited the XRD patterns of Fe_3_O_4,_ g-C_3_N_4_ and Fe_3_O_4_-g-C_3_N_4_-TCT-PAA-Ag nanocomposite. The comparison of the XRD pattern of the catalyst with that of Fe_3_O_4_ showed that all the characteristic peaks of Fe_3_O_4_ nanoparticle i.e. 30.2°, 35.5°, 43.1°, 53.4°, 57.1°, and 62.8°, relating indices (2 2 0), (3 1 1), (4 0 0), (4 2 2), (5 1 1), and (4 4 0), are observed in the XRD pattern of Fe_3_O_4_-g-C_3_N_4_-TCT-PAA-Ag. These peaks can be indexed to a face-centered cubic structure with the Fd3m space group of Fe_3_O_4_ correspondent to JCPDS card no. 19‐0629. Moreover, it can be observed that the XRD pattern of the catalyst exhibited the characteristic band of g-C_3_N_4,_ i.e., a strong peak in the diffractogram at 2θ = 28° (JCPDS card no. 87–1526). XRD pattern of crystalline planes of cubic Ag gave four characteristic crystalline peaks at 2θ = 38.0°, 44.3°, 64.4°, and 77.5° that could be attributed to the reflections of the (1 1 1), (2 0 0), (2 2 0) and (3 1 1) (JCPDS card no. 65‐2871).

**Figure 4:**
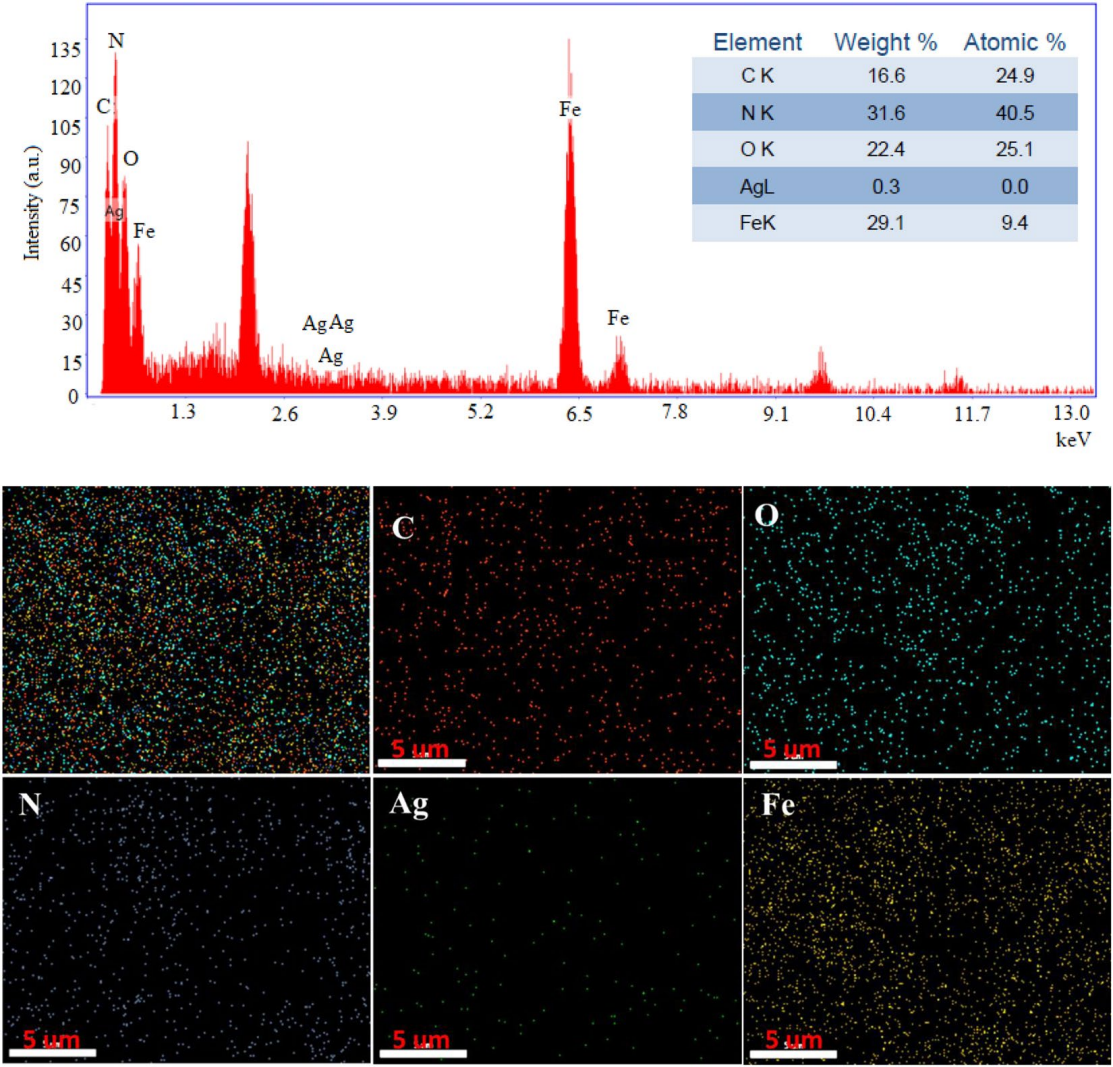
EDX-Mapping analysis of Fe_3_O_4_-g-C_3_N_4_-TCT-PAA-Ag nanocomposite.

To evaluate the elemental composition and distribution of nanoparticles on the synthesized nanocomposite, EDX and elemental mapping analysis were performed, Fig. 4. The study results showed that besides the C and N elements, the Ag, Fe, and O elements are identified. The mapping analysis, Fig. 4 demonstrated the successful loading of Ag and Fe_3_O_4_ nanoparticles onto the g-C_3_N_4_ surface. This analysis also illustrated the uniform dispersion of these nanoparticles on the g-C_3_N_4_ substrate.

**Figure 5:**
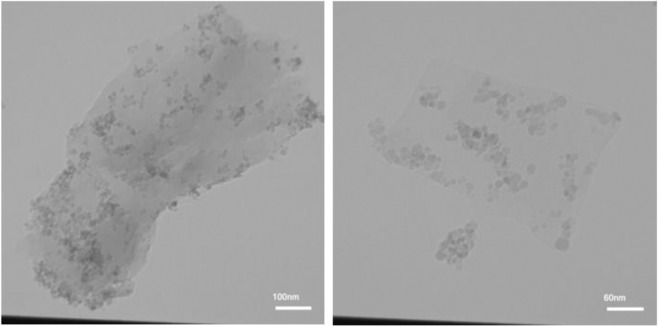
TEM images of Fe_3_O_4_-g-C_3_N_4_-TCT-PAA-Ag nanocomposite.

To obtain information about the morphology, TEM analysis was performed. The TEM images of Fe_3_O_4_-g-C_3_N_4_-TCT-PAA-Ag are demonstrated in Fig. 5. The g-C_3_N_4_ thin film is observed, which is decorated by the Ag and Fe_3_O_4_ nanoparticles (black spots). As shown, Fe_3_O_4_ and Ag nanoparticles were almost uniformly dispersed on g-C_3_N_4_.

**Figure 6:**
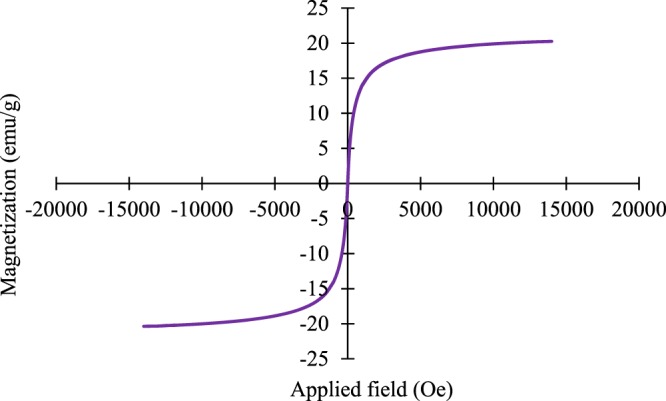
VSM of Fe_3_O_4_-g-C_3_N_4_-TCT-PAA-Ag nanocomposite.

The evaluation of the magnetic properties of Fe_3_O_4_-g-C_3_N_4_-TCT-PAA-Ag nanocomposite was performed using the VSM technique at room temperature. The VSM plot of Fe_3_O_4_-g-C_3_N_4_-TCT-PAA-Ag nanocomposite is shown in Fig. 6. The appraised value of Saturation magnetization (Ms) was 20.22 emu/g, indicated that the catalyst has paramagnetic behavior. This result showed the synthesized nanocomposite could be simply separated with the help of an external magnet (Fig. 6). This confirmed that the magnetic property of Fe_3_O_4_ nanoparticles was partially maintained even after its composition with Ag nanoparticle, g-C_3_N_4_, and polyallylamine.

**Figure 7:**
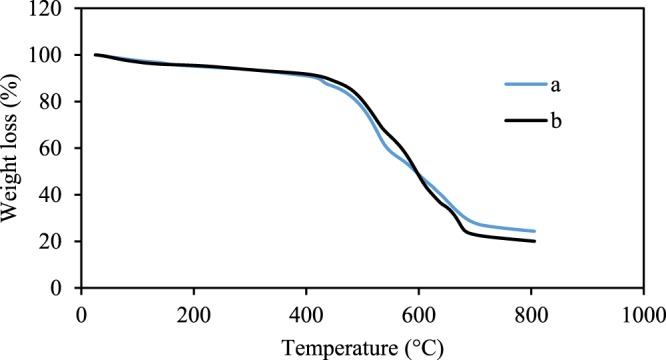
TGA of (**a**) Fe_3_O_4_-g-C_3_N_4_-TCT and (**b**) Fe_3_O_4_-g-C_3_N_4_-TCT-PAA-Ag nanocomposite.

Thermal properties of the synthesized nanocomposite before and after the formation of polymer were assessed by the TGA technique, as exhibited in Fig. 7. The degradation of nanocomposite occurred in several stages. A slight weight loss was observed at a temperature below 200 °C due to the loss of the adsorbed water. The subsequent mass loss of about 75.61% in the curve a (Fe_3_O_4_-g-C_3_N_4_-TCT), was related to the loss of organic compounds in this structure. In the curve b (Fe_3_O_4_-g-C_3_N_4_-TCT-PAA-Ag), apart from the weight loss due to the loss of water, an additional weight loss of 79.77% was observed. Comparing the two cures, it can be inferred that the polyallylamine content was ~3 wt%.

### Evaluation of the catalytic performance

**Figure 8:**
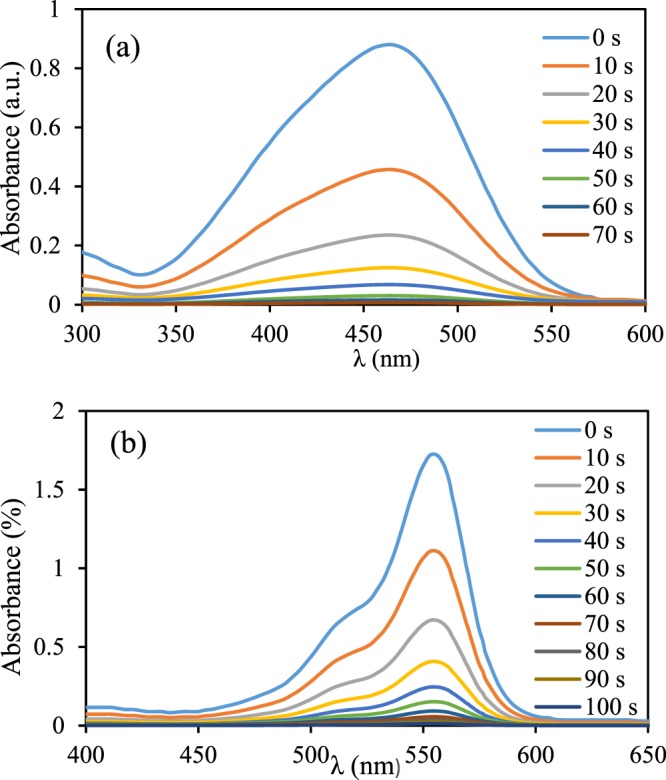
UV-Vis spectra for reduction of MO (**a**), and RhB (**b**) using Fe_3_O_4_-g-C_3_N_4_-TCT-PAA-Ag nanocatalyst.

MO and RhB as common contaminants were chosen to investigate the catalytic efficiency of the Fe_3_O_4_-g-C_3_N_4_-TCT-PAA-Ag with sodium borohydride as a reducing agent. In the lack of the Fe_3_O_4_-g-C_3_N_4_-TCT-PAA-Ag, the reduction with NaBH_4_ was deficient, and after 3 h, no notable variations in the concentration of dyes were apperceived (Conversion = 2%). Furthermore, in the presence of Fe_3_O_4_-g-C_3_N_4_-TCT-PAA-Ag without NaBH_4_, no considerable change in the concentration of dyes was observed (Conversion ~ 0%). These observations showed that neither nanocatalyst nor NaBH_4_ was able to reduce these dyes individually. Therefore, both Fe_3_O_4_-g-C_3_N_4_-TCT-PAA-Ag nanocomposite and sodium borohydride were required in the catalytic reduction of MO and RhB. As presented in Fig. 8, when the reduction reactions of MO and RhB with a given amounts of the Fe_3_O_4_-g-C_3_N_4_-TCT-PAA-Ag nanocomposite was initiated, the absorption peak at 460 nm for MO and 556 nm for RhB gradually decreased in intensity.

The effect of the amount of Fe_3_O_4_-g-C_3_N_4_-TCT-PAA-Ag (1, 2, 3, 4, and 5 mg) on the catalytic performance in the reduction of dyes was investigated. As expected, the catalytic activity of Fe_3_O_4_-g-C_3_N_4_-TCT-PAA-Ag was enhanced by increasing the nanocatalyst amount. As exhibited in Table S1, the low amount of nanocatalyst in a short time was required for the reduction of two dyes. The best results were obtained with 2.0 mg of the Fe_3_O_4_-g-C_3_N_4_-TCT-PAA-Ag for MO and 4.0 mg of this nanocomposite for RhB in the presence of fresh NaBH_4_ solution (0.1 M) at ambient temperature.

The variation of ln(A_t_/A_0_) vs. reaction time was linear; indicating that the reaction follows Langmuir-Hinshelwood model with pseudo-first-order kinetics, where A_0_ and A_t_ were the absorbance of these dyes after time 0 and t, respectively. As ln(C_t_/C_0_) = ln (A_t_/A_0_) = −k.t, the k (apparent rate constant) was obtained from the slope of this lines. These constants for MO and RhB are reported in Fig. S1 and Table 1 at four different temperatures.

**Table 1:** The results of thermodynamic and kinetic parameters of reduction reaction of MO and RhB in the presence of the Fe3O4-g-C3N4-TCT-PAA-Ag nanocatalyst.

Dye	T (K)	k (s^−1^)	Ea (KJ/mol)	∆S (J/mol.K)	∆H (KJ/mol)
MO	298 303 308 313	0.045 0.050 0.067 0.101	36.72	−43.22	38.51
RhB	298 303 308 313	0.041 0.067 0.080 0.085	44.55	−16.61	47.13

### Thermodynamic study

The activation energies of RhB and MO reduction reactions at four various temperatures were determined by the Arrhenius equation (ln k = ln A– (E_a_/RT)). In this equation, A is the Arrhenius factor, E_a_ is the activation energy, R is the ideal gas constant (8.314 JK^−1^ mol^−1^), and T is temperature. From drawing the graphs of lnk vs 1/T, the activation energy values were obtained as 36.72 and 44.55 KJ mol^−1^ for MO and RhB, respectively, as exhibited in Fig. S2 and Table 1.

The thermodynamic parameters i.e. activation enthalpy (∆H^#^) and activation entropy (∆S^#^) were measured by Eyring equation (ln (k/T) = ln (k_B_/h) + ∆S^#^/R – ∆H^#^/R (1/T)). Where, k_B_ and h are the Planck constant (6.626 × 10^−34^ J K^−1^ mol^−1^) and the Boltzmann constant (1.381 × 10^−23^ J K^−1^). Figure S3 demonstrated the plot of ln(k/T) vs. 1/T for the MO and RhB reduction reaction obtained for different temperatures. The amounts of enthalpy for the MO and RhB reactions catalyzed by Fe_3_O_4_-g-C_3_N_4_-TCT-PAA-Ag nanocomposite were calculated as 38.51 and 47.13 kJ mol^−1^ respectively. The values of entropy for MO and RhB reduction, were obtained as −43.22 and −16.61 J mol^−1^ K^−1^, respectively, as given in Table 1.

### Mechanism of reduction of dyes by Fe_3_O_4_-g-C_3_N_4_-TCT-PAA-Ag nanocatalyst

**Figure 9:**
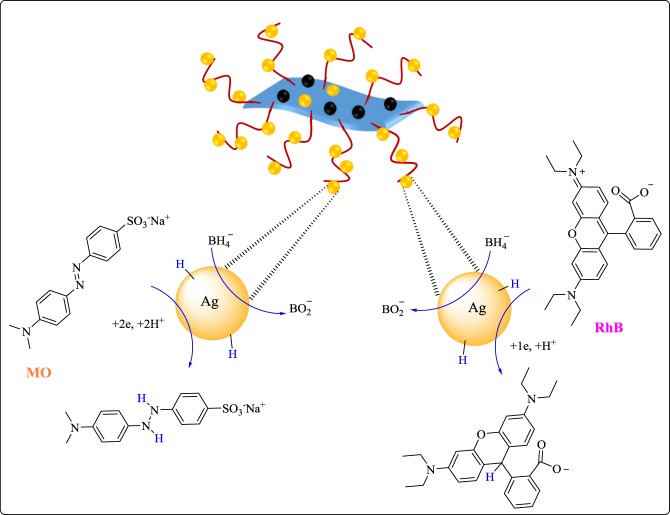
The plausible mechanism for the reduction of dye in the presence of Fe_3_O_4_-g-C_3_N_4_-TCT-PAA-Ag.

The reduction of MO and RhB dyes in the presence of Fe_3_O_4_-g-C_3_N_4_-TCT-PAA-Ag nanocatalyst and sodium borohydride is as follows, according to the literature^35^, in the first step, dye as H^−^ acceptor, and BH_4_^−^ molecules as H^−^ donor are adsorbed on the surface of the nanocatalyst via the electrostatic interaction, hydrogen bonding, and π-π interaction. In other words, these molecules transfer from the solution to the nanocatalyst surface. Then, electrons transfer from BH_4_^−^ to the organic dyes, MO or RhB, and this process leads to the decolorization and reduction of dyes (Fig. 9). The formed product desorbed from the Fe_3_O_4_-g-C_3_N_4_-TCT-PAA-Ag surface and diffused from the surface to the solution region. Therefore, it can be accepted that the catalytic system is a requirement for performing the MO or RhB reduction because it overcomes the kinetic barrier and catalyzes the reduction reaction by simplifying the electron transition between electron donor i.e. BH_4_^−^ and electron acceptor i.e. dye, as a result, the reduction and decolorization of dye occurred. The cycle again continues after the discharge of the active sites by desorption of the products.

### Recyclability

**Figure 10:**
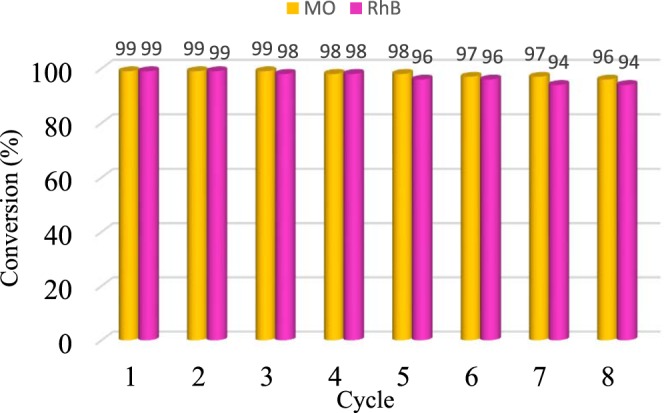
The recyclability of the nanocatalyst for the reduction of MO and Rh B under optimum reaction condition.

Encouraged by high efficiency of the catalyst for dye reduction, the stability and reusability of the Fe_3_O_4_-g-C_3_N_4_-TCT-PAA-Ag nanocatalyst were investigated. Recycling was repeated for eight times for reduction of MO and RhB dyes. For each individual cycle, the nanocatalyst was separated with the help of an external magnet from the reaction solution. The separated nanocatalyst was then rinsed with water and ethanol several times, dried, and applied for the subsequent run. It was observed that the catalytic activity of Fe_3_O_4_-g-C_3_N_4_-TCT-PAA-Ag remained almost constant up to 8 cycles for dye reduction reactions. In more detail, MO conversion decreased from 99 to 96 and the loss of the catalytic activity of the catalyst for RhB reduction was 5% after eight reaction runs (Fig. 10). To study Ag leaching, ICP technique was applied. The amount of Ag loaded on the Fe_3_O_4_-g-C_3_N_4_-TCT-PAA-Ag catalyst after 8 cycles was obtained as 0.32 wt%, which was slightly lower compared to the fresh catalyst (0.34wt %).

Figure S4 illustrated the FTIR spectrum of Fe_3_O_4_-g-C_3_N_4_-TCT-PAA-Ag nanostructures recycled after catalytic cycles along with that of fresh RhB. The similarity of two spectra confirmed that Fe_3_O_4_-g-C_3_N_4_-TCT-PAA-Ag preserved its structure and recycling did not destruct it.

## Experimental

### Materials

Silver nitrate (AgNO_3_, 99%), iron(III) chloride hexahydrate (FeCl_3_.6H_2_O, 97%), iron(II) chloride tetrahydrate (FeCl_2_.4H_2_O, 99%), sodium borohydride (NaBH_4_, 96%), melamine (C_3_H_6_N_6_, 99%), ammonium hydroxide solution (NH_4_OH, 25%), cyanuric chloride (2,4,6-trichloro-1,3,5-triazine, C_3_Cl_3_N_3_, TCT, 99%), azobisisobutyronitrile (AIBN, (CH_3_)_2_C(CN)N = NC(CH_3_)_2_CN, 98%), rhodamine B (C_28_H_31_ClN_2_O_3_, 95%), methyl orange (C_14_H_14_N_3_NaO_3_S, 85%), and allylamine (CH_2_ = CHCH_2_NH_2_, 99%) were purchased from Sigma-Aldrich. The detail of the used instruments for the characterization of the catalyst is reported in SI.

### Heracleum persicum extract preparation

*Heracleum persicum* herb was collected and entirely washed with distilled water. After drying, in 100 mL of distilled water, 10 g of the grass was added, and using Soxhlet, the extraction process was performed. After the completion process, the extract was dried to get *Heracleum persicum* extract. The extract was stored at 4 °C.

### Synthesis of Fe_3_O_4_-g-C_3_N_4_-TCT-PAA-Ag nanocomposite

Synthesis of Fe_3_O_4_-g-C_3_N_4_-TCT-PAA-Ag nanocomposite includes several stages:

#### Synthesis of Fe_3_O_4_-g-C_3_N_4_

Typically, 1.5 g of g-C_3_N_4_ was dispersed in 140 mL of distilled water, and then 1.37 g of FeCl_3_.6H_2_O and 0.5 g of FeCl_2_.4H_2_O were added to it. This mixture was heated at 60 °C. Next, 11 mL of NH_4_OH solution (25%) was added to the above mixture. The reaction mixture was stirred for another 60 min, and then it was cooled to ambient temperature. After separating of the magnetic nanostructures with an external magnetic, the product was washed several times with water and dried at room temperature.

#### Synthesis of Fe_3_O_4_-g-C_3_N_4_-PAA

Fe_3_O_4_-*g-*C_3_N_4_ was dispersed in dried tetrahydrofuran (THF) (25 mL) and stirred for 20 min at 0–5 °C. Then a solution of cyanuric chloride (2 mmol in 25 mL THF) was transferred to the abovementioned mixture. The suspension was stirred for 4 h. At the completion of this reaction, the product was separated magnetically and rinsed with THF frequently. In the next step, the resulting product was dispersed in THF (50 mL), and 3 mmol allylamine was added. The mixture was stirred and heated at 80 °C for 5 h. After the end of this reaction, the precipitate was isolated from the reaction mixture using a magnet and rinsed with ethanol. To form PAA polymer, the resulting product was dispersed in EtOH (30 mL) and 10 mmol allylamine and AIBN as an initiator of radical polymerization were added. The mixture was stirred and refluxed overnight. The product was isolated using a magnet, rinsed with ethanol, and dried under ambient condition.

#### Synthesis of Fe_3_O_4_-g-C_3_N_4_-TCT-PAA-Ag

300 mg of Fe_3_O_4_-g-C_3_N_4_-PAA nanocomposite was dispersed to 50 mL of AgNO_3_ (1.5 mM) solution, then, 10 mL of *Heracleum persicum* extract (2%) was added to the abovementioned solution under stirring at 60 °C for 2 h. After cooling the reaction mixture, the Fe_3_O_4_-g-C_3_N_4_-TCT-PAA-Ag was separated with an external magnet, washed several times with ethanol/water, and dried. Using ICP analysis, the content of Ag was estimated as 0.34wt %

### Reduction of dyes using Fe_3_O_4_-g-C_3_N_4_-TCT-PAA-Ag catalyst

The evaluation of the catalytic performance of the synthesized nanocomposite was performed in a quartz cell. MO aqueous solution (10 ppm) and freshly prepared NaBH_4_ solution (0.1 M) were taken in quartz cell. Then, Fe_3_O_4_-g-C_3_N_4_-TCT-PAA-Ag nanocatalyst (2.0 mg) was added into the above mentioned solution. The progress of the catalytic reduction reaction was monitored by recording the time-dependent spectra with a UV–Vis spectrophotometer. In order to investigate catalyst recycling, the nanocatalyst was separated by an external magnet, washed, and reused for consequent reactions with fresh dye solutions. Also, the same process was performed for RhB except that the amount of nanocatalyst was 4.0 mg.

## Conclusion

In this work, eco-friendly and green synthesis of Ag nanoparticle was reported using *Heracleum persicum* extract as a reducing and stabilizing agent. The nanoparticles were supported on the magnetic polyallylamine decorated g-C_3_N_4_ substrate to furnish magnetic heterogeneous Fe_3_O_4_-g-C_3_N_4_-TCT-PAA-Ag. It was found that Fe_3_O_4_-g-C_3_N_4_-TCT-PAA-Ag could efficiently catalyze the reduction of MO and Rh B dyes, within 70 s and 100 s respectively, in the presence of the NaBH_4_ solution as a reducing agent. Using the experimental data, E_a_, ΔH^#^, and ΔS^#^ values for reductive degradation of MO were calculated as 36.72 kJ mol^−1^, 38.51 kJ mol^−1^, and −43.22 J mol^−1^ K^−1^, respectively. These values for RhB were measured as 44.55 kJ mol^−1^, 47.13 kJ mol^−1^, and −16.61 Jmol^−1^K^−1^, respectively. The results of recyclability of Fe_3_O_4_-g-C_3_N_4_-TCT-PAA-Ag confirmed high recyclability of the catalyst (up to eight reaction runs).

## Supplementary information

Supplementary information.
