# Supplementary material for: Green synthesis of Ag NPs on magnetic polyallylamine decorated g-C3N4 by Heracleum persicum extract: efficient catalyst for reduction of dyes
Source: Sci Rep. 2020 Apr 20;10:6579. doi: 10.1038/s41598-020-63756-4 (PMC7171167; doi:10.1038/s41598-020-63756-4)
Supplement: Supplementary file 1 — Supplementary information. [file 41598_2020_63756_MOESM1_ESM.docx]

**Supporting information**

**Green synthesis of Ag NPs on magnetic polyallylamine decorated g-C_3_N_4_ by *Heracleum persicum* extract: efficient catalyst for reduction of dyes**

Pourya Mohammadi^1^, Majid M Heravi^1^,* Samahe Sadjadi^2^ *

**Instrumentation**

The size, morphology, and element composition of Fe_3_O_4_-g-C_3_N_4_-TCT-PAA-Ag nanocomposite were investigated by TEM and energy-dispersive X-ray (EDX) technique. These data were obtained from Philips CM30 microscope operated at 300 Kv and TESCAN MIRA III microscope. X-ray diffraction (XRD) pattern was performed on a PHILIPS PW1730 diffractometer with Cu K_α_ radiation (1.54056 A) working at 40 kV. The absorption spectra of dyes were recorded using a UV–Visible Spectrophotometer (PerkinElmer, Lambda 365). TG analysis was carried out on a TA Q600 instrument under the nitrogen atmosphere at a heating rate of 10 °C min^-1^. The functional groups present in the prepared nanocomposite were recognized by using Thermo AVATAR FTIR Spectrophotometer. A Lakeshore7407 magnetometer was used for the measurement of magnetic properties of the synthesized nanocomposite at room temperature.

**Table S1.** The optimization of the Fe_3_O_4_-g-C_3_N_4_-TCT-PAA-Ag amount for reduction of MO and RhB

| Dye | Catalyst amount (mg)^a^ | Conversion (%) |
| --- | --- | --- |
| MO | 1 | 69 |
|  | 2 | 99 |
|  | 3 | 99 |
|  | 1 | 56 |
|  | 2 | 68 |
| RhB | 3 | 82 |
|  | 4 | 99 |
|  | 5 | 99 |

**Figure S1.** The plot of ln (A_t_/A_0_) vs. time (s) for reduction of MO (a) and RhB (b)

**Figure S2.** The plot of lnk vs. 1/T for reduction of MO (a) and RhB (b).

**Figure S3.** The diagram of ln k/T vs. 1/T for reduction of MO (a) and RhB (b).

**Figure S4.** The FTIR spectra of fresh and reused Fe_3_O_4_-g-C_3_N_4_-TCT-PAA-Ag after eight reaction runs
